# Validation of MLC leaf open time calculation methods for PSQA in adaptive radiotherapy with tomotherapy units

**DOI:** 10.1002/acm2.14478

**Published:** 2024-08-08

**Authors:** Marie Nasrallah, François Bochud, Neelima Tellapragada, Jean Bourhis, Edward Chao, Dylan Casey, Raphaël Moeckli

**Affiliations:** ^1^ Institute of Radiation Physics Lausanne University Hospital and Lausanne University Lausanne Switzerland; ^2^ Accuray Incorporated Madison Wisconsin USA; ^3^ Radiation Oncology Department Lausanne University Hospital and Lausanne University Lausanne Switzerland

**Keywords:** adaptive radiation therapy (ART), leaf open time (LOT), patient specific quality assurance (PSQA), tomotherapy

## Abstract

**Background:**

Treatment delivery safety and accuracy are essential to control the disease and protect healthy tissues in radiation therapy. For usual treatment, a phantom‐based patient specific quality assurance (PSQA) is performed to verify the delivery prior to the treatment. The emergence of adaptive radiation therapy (ART) adds new complexities to PSQA. In fact, organ at risks and target volume re‐contouring as well as plan re‐optimization and treatment delivery are performed with the patient immobilized on the treatment couch, making phantom‐based pretreatment PSQA impractical. In this case, phantomless PSQA tools based on multileaf collimator (MLC) leaf open times (LOTs) verifications provide alternative approaches for the Radixact® treatment units. However, their validity is compromised by the lack of independent and reliable methods for calculating the LOT performed by the MLC during deliveries.

**Purpose:**

To provide independent and reliable methods of LOT calculation for the Radixact® treatment units.

**Methods:**

Two methods for calculating the LOTs performed by the MLC during deliveries have been implemented. The first method uses the signal recorded by the build‐in detector and the second method uses the signal recorded by optical sensors mounted on the MLC. To calibrate the methods to the ground truth, in‐phantom ionization chamber LOT measurements have been conducted on a Radixact® treatment unit. The methods were validated by comparing LOT calculations with in‐phantom ionization chamber LOT measurements performed on two Radixact® treatment units.

**Results:**

The study shows a good agreement between the two LOT calculation methods and the in‐phantom ionization chamber measurements. There are no notable differences between the two methods and the same results were observed on the different treatment units.

**Conclusions:**

The two implemented methods have the potential to be part of a PSQA solution for ART in tomotherapy.

## 1 INTRODUCTION

The achievement of a safe and accurate dose delivery is one of the main concerns in radiation therapy (RT). Among others, national and international guidelines recommend performing patient specific quality assurance (PSQA) to identify potential discrepancies between the dose calculated by the treatment planning system (TPS) and the dose delivered by the treatment delivery system (TDS).^1^, ^2^, ^3^, ^4^

Many methods of PSQA exist, involving electronic portal imaging device (EPID), film dosimetry, ionization chamber measurements, diode array dosimetry, and secondary dose calculation.^4^ Usually, the PSQA workflow consists of recalculating the dose planned by the TPS on a phantom geometry and irradiating the phantom according to the patient treatment plan. The delivered dose measured in the phantom is compared to the TPS predicted dose. The gamma index metric is generally used for dose comparison.^5^ Although this PSQA process is commonly implemented in clinical workflows, it has some drawbacks as it stands. In fact, a phantom‐based PSQA implies dedicated time to be allocated to do the setup and the measurements. Secondly, this process only performs pretreatment verifications and does not verify the treatment fraction delivery quality itself. Thus, it does not detect errors that could happen during the fraction delivery such as patient anatomy changes, machine delivery errors, incorrect plan delivery, or accidental plan modification.^6^, ^7^ Finally, many studies reported that the gamma metric might fail to detect or emphasize clinically relevant errors.^8^, ^9^, ^10^, ^11^, ^12^, ^13^, ^14^, ^15^, ^16^, ^17^

The emergence of online adaptive radiation therapy (ART) adds new complexities to the dose delivery verification. In online ART workflow, the treatment may be adapted from fraction to fraction to take into account significant daily anatomical changes. Organ at risks and target volume re‐contouring as well as plan re‐optimization and treatment delivery are performed while the patient remains immobilized on the treatment couch. Consequently, it is not possible to perform delivery verifications based on pretreatment phantom measurements and it becomes challenging to perform pretreatment PSQA. To overcome this complexity, some studies investigated the possibility to use predictive secondary dose calculations,^18^ periodic dose measurements,^19^ machine learning models,^20^, ^21^ or statistical methods.^22^

Tomotherapy is a specific modality for intensity modulation radiation therapy (IMRT). In tomotherapy, the beam intensity is modulated by adjusting the leaf open times (LOTs) of the multileaf collimator (MLC).^23^

It has been shown that deviations between planned and actual MLC (LOTs) may lead to clinically relevant dosimetric inaccuracies in tomotherapy.^24^, ^25^ Some studies proposed PSQA techniques based on MLC LOTs verifications.^25^, ^26^, ^27^ Although they give an alternative for PSQA in ART, their validity is compromised by the lack of independent and reliable methods for calculating the LOT performed by the MLC during deliveries. They either use a LOT calculation method provided by the vendor, which makes it not independent, or they use an independent method without verifying its validity against the ground truth.

The aim of this work is to provide independent methods for LOT calculation and to verify their validity against the ground truth provided by in‐phantom ionization chamber measurements. For this purpose, two methods of LOT calculation were implemented. The first one was developed by Schopfer et al.^28^ and uses MegaVoltage (MV) detector data and the second one was developed in this work and uses leaf optical sensor data to calculate the LOTs. In this work, both methods were calibrated according to in‐phantom ionization chamber LOT measurements performed on a Radixact treatment unit located at Accuray Inc. factory. To validate the methods, the calculated LOTs were compared to in‐phantom ionization chamber LOT measurements performed on two treatment units at our institute. Although the MV detector, the optical sensor, and the ionization chamber methods involve both measurements and calculations, for the sake of convenience, the first two methods are referred to as “calculations” and the ionization chamber method is termed “measurements” in this study.

## 2 MATERIALS AND METHODS

### 2.1 Tomotherapy unit and planned LOTs

Tomotherapy delivers intensity modulation radiation therapy (IMRT) treatments with a fan beam and a multileaf collimator (MLC) composed of 64 leaves to modulate the beam intensity. The leaves are driven by compressed air to allow fast transitions between closed and open states. The rotational delivery is segmented into 51 projections, providing the capability to specify the amount of time during which individual leaves stay open within each segment (leaf open time, LOT). The configuration of the LOTs throughout the treatment is determined by the planned LOT sinogram. This consists of a data array with one row per leaf and one column per projection, each pixel containing the planned LOT for the corresponding leaf and projection.^28^, ^29^

Tomotherapy units use jaws to set the field width along the patient's longitudinal direction. The jaws opening is preset to 1, 2.5, or 5 cm field width at half maximum (FWHM) in longitudinal direction at isocenter. The 2.5 and 5 cm opening modes can operate with static jaws or with dynamic jaws, which adapt the field width during the treatment to reduce the penumbras at cranial and caudal target edges. The 1 cm opening mode uses static jaws.

The data used in this study were collected from three different Radixact units. The first one was used for calibration and is located at Accuray Inc. factory (called T4), the two others were used for verification and are located at our institute (called Tomo1 and Tomo2, respectively).

### 2.2 LOT calculation with the MV detector data

A method developed by Schopfer et al.^28^ uses the signal recorded by the MV detector during deliveries to calculate the performed LOTs. The MV detector is mounted on the gantry ring opposite to the linear accelerator (LINAC) and comprises 640 adjacent ionization chambers. It records the exit fluence per linac pulse with a sample rate of 300 Hz. The method workflow is summarized in the following section and detailed in Schopfer et al.^28^

**FIGURE 1:**
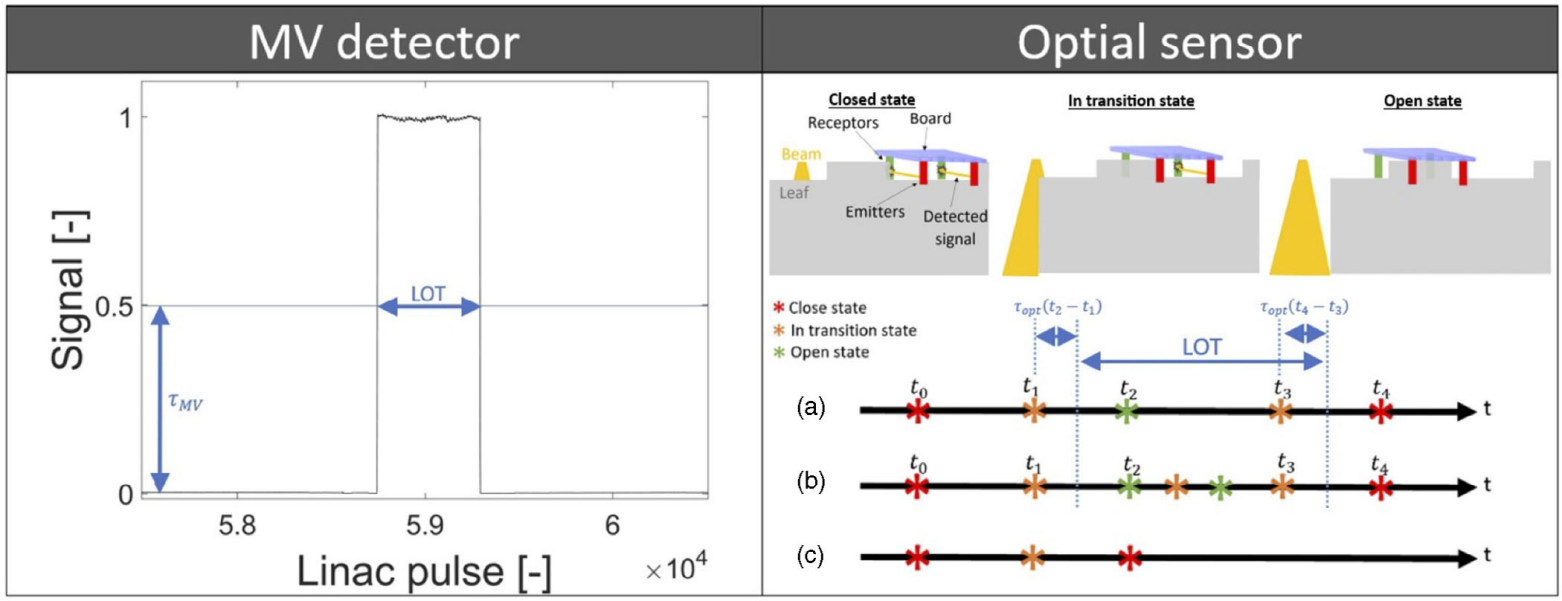
LOT measurement with MV detector data (left) and optical sensor data (right). The optical sensors detect three different leaf transition sequences: (a) Normal LOT transition sequence, (b) Short LOT (below 50 ms) transition sequence, and (c) short leaf closed time transition sequence.

Firstly, the MV detector signal is preprocessed. The signal is corrected where electrical arcing occurred. The signal is interpolated related to leaf‐to‐channel‐mapping to link each leaf to one ionization chamber of the MV detector. The signal offset caused by the MLC leakage radiation and the detector dark current is subtracted from the signal. The signal is deconvolved with the Richardson–Lucy deconvolution algorithm to reduce inter‐leaf signal contamination. Then, the MV detector signal amplitude is normalized by the maximum signal value near projection center for each leaf and each projection. After normalization, the leaf signal amplitude is close to 1 for a fully open leaf (as opposed to a leaf, which is transitioning from closed to open states) and close to 0 for a fully closed leaf. Secondly, the actual LOTs are calculated for each individual leaf by measuring the width of the corresponding signal segment at height $\tau_{MV}$ (Figure 1). The value of $\tau_{MV}$ is discussed in Section 2.5.

Accuray Inc. used an MV detector method for earlier generation delivery systems.

### 2.3 LOT calculation with the leaf position sensors data

A method which uses optical sensors mounted on the MLC to calculate the performed LOTs was developed. These sensors are used to monitor leaf positions during deliveries (Figure 1). Two photoemitters are positioned on one side and two photoreceptors on the other side of each leaf. The photodiodes are fixed on a static board positioned over the leaf. Each leaf contains a notch used to keep track of the leaf position with respect to the photodiode positions. When the leaf blocks the treatment beam, the photodiodes are aligned with the notch and the two photoreceptors receive signal from the photoemitters, the leaf is in closed state. When the leaf partially blocks the beam, only one photodiode is aligned with the notch and detects signal, the leaf is in transition state. Finally, the leaf is in open state when the leaf does not block the beam, the two photodiodes are not aligned with the notch and no signal is detected. The states of the optical sensors are continuously read with a cycle time of approximately 0.18 ms (5556 Hz). The states of all the leaves are recorded each time one leaf is changing state.

A moving leaf can undergo three different state sequences (Figure 1). In the prevailing sequence, the leaf opens and closes by passing through “in transition” states (Figure 1a). An alternative sequence happens when the commanded time between two “open” states is such small (below 50 ms) that the leaf does not have time to close fully before opening again. In this case, no “close” state is observed between two open events (Figure 1b).

A method of LOTs calculation based on the optical sensor data has been developed in this work. For this purpose, the LOT is defined as the time interval between the leaf opening and the leaf closing. However, during in transition states the leaf is neither fully open nor fully closed. To overcome this complexity, the optical sensor threshold, denoted $\tau_{opt}$, should be introduced. This threshold is used to determine where the leaf is considered open and where it is considered closed in the transition state. To establish this threshold, ionization chamber measurements were performed (see Section 2.5).

LOTs based on the optical sensor measurements are calculated with Equation (1)

(1) $$\begin{array}{c} \;LOT_{opt}=t_{3}+\tau_{opt}\left(t_{4}-t_{3}\right)-\left(t_{1}+\tau_{opt}\left(t_{2}-t_{1}\right)\right) \end{array}$$

where $t_{1}$ is the time when the leaf changes from “closed” to “in transition” state, $t_{2}$ the time when the leaf changes from “in transition” to “open” state, $t_{3}$ the time when the leaf changes from “open” to “in transition” state, $t_{4}$ the time when the leaf changes from “in transition” to “close” state, and $\tau_{opt}$ is the optical sensor threshold.

Another possible sequence happens when the commanded LOT is so small that the leaf does not have time to open fully. In this case, no “open” state is observed and the LOT is set to 0 (Figure 1c).

We used MATLAB® R2021a (MathWorks, Inc.) for the implementation of the optical sensor LOT calculation method.

Accuray Inc. currently uses the optical sensor method for MLC performance evaluation.

### 2.4 Reference plans

**FIGURE 2:**
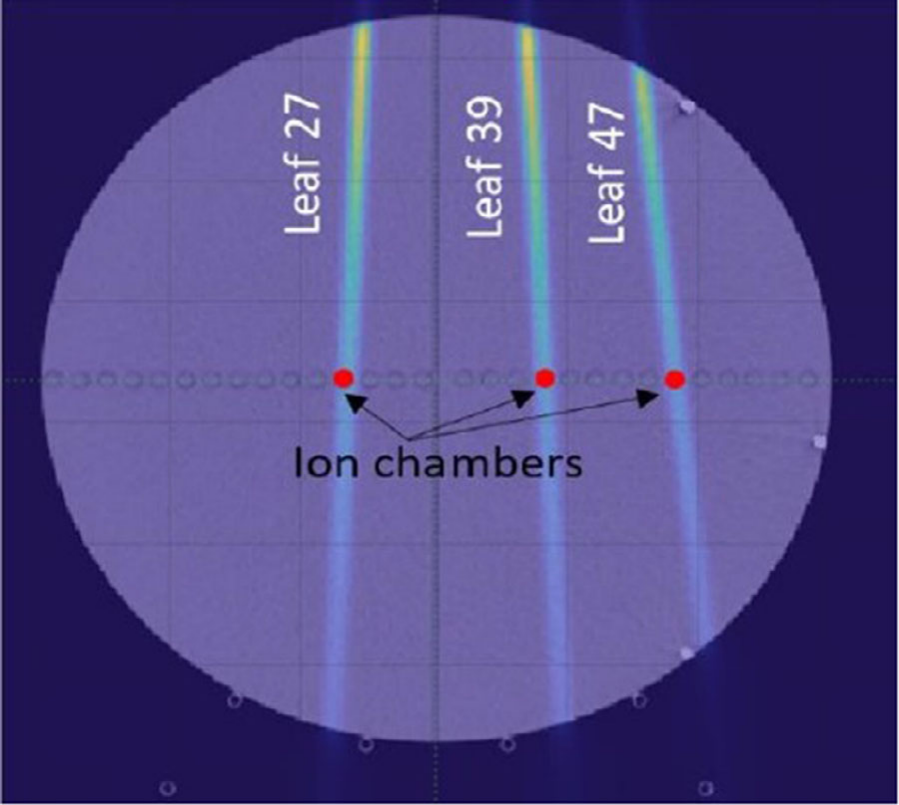
Transversal view of the cheese phantom used for the measurements. Three A1SL ionization chambers were inserted in the phantom on the beamlet paths of leaves 27, 39, and 47.

Actual LOTs performed by the MLC during the deliveries, referred to as the “ground truth,” have to be known in order to validate both techniques described above. Therefore, a method of LOTs measurements with ionization chambers was developed. For this purpose, A1SL ionization chambers (Standard Imaging, USA) were placed within a solid water phantom allowing the incorporation of inserts, called the cheese phantom (Figure 2).

Twelve plans with static gantry at 0° angle, static couch, 400 projections, and a time per projection of $t_{proj}=348\;\mathrm{ms}$ were generated. Additionally, the leaves 27, 39, and 47 were active while the other leaves remained closed during the whole delivery. Four plans per jaw opening (JO) of 1, 2.5, and 5 cm were generated, each plan with a different active leaves LOT of 25%, 50%, 75%, and 100% of the projection time.

### 2.5 Calibration of the LOT calculation methods

There is no consensus on the thresholds which should be used to calculate the LOTs with the MV detector method. Some authors defined the LOTs as the full width at half maximum ($\tau_{MV}=0.50)$ of the normalized signal^27^, ^30^ or as the sum of the products of final fluence and pulse length at each projection.^31^ Concerning the optical sensor method, Accuray Inc. uses the threshold $\tau_{opt}=0.50$. Ionization chamber measurements were performed to determine the LOTs actually performed by the MLC during the deliveries. That “ground truth” was compared with the two techniques and the corresponding thresholds were determined.

Each plan described in Section 2.4 were delivered twice on a cheese phantom with the T4 Radixact unit. After each delivery, the charges measured by the ionization chambers were collected and the procedure raw data file was retrieved from the treatment delivery console.

The charges measured by the ionization chambers were then converted into LOTs. For this purpose, the mean LOT performed by the leaf which produces a beamlet detected by the ionization chamber *i* is calculated with Equation (2):

(2) $$\begin{array}{c} LOT_{x}\;\left(i,jo\right)=\frac{q_{x}\left(i,jo\right)}{q_{100\%}\left(i,jo\right)}\;t_{proj} \end{array}$$

where $q_{x}\left(i,jo\right)$ is the charge measured by the ionization chamber i for the plan with jaw opening jo of 1, 2.5, or 5cm and LOTs of x=25%, x=50%, or x=75% of the projection time, $q_{100\%}\left(i,jo\right)$ is the charge measured by the ionization chamber i for the plan with jaw opening jo and active leaves LOTs of 100% of the projection time and $t_{proj}=348\;\mathrm{ms}$ is the projection time.

The MV detector and the optical sensor data collected in the raw data files were used to calculate the mean LOTs performed by the active leaves for the different plans. For both LOT calculation methods, the measured thresholds were found by comparing the results of the methods with the ground truth.

### 2.6 Validation of the LOT calculation methods

To analyze the results obtained during calibration process, the discrepancies between the mean LOTs measured with the optical sensor, MV detector and ion chamber methods, and the planned mean LOTs were calculated with the thresholds $\tau_{opt}=0.50$ and $\tau_{MV}=0.50$ as well as with the measured thresholds. To verify the validity of the measured thresholds, a *t*‐test statistical analysis was performed between the measured and the calculated mean LOT discrepancies for both LOT calculation methods with the different thresholds.

To analyze the validity of the LOT calculation methods and of the measured thresholds across different treatment machines, the measurements were reproduced on the Tomo1 and Tomo2 Radixact units. Each plan described in Section 2.4 was delivered twice on a cheese phantom (Figure 2). The charges measured by the ionization chambers were converted into mean LOTs with Equation (2). MV detector and the optical sensor data collected in the raw data files were used to calculate the mean LOTs performed by the active leaves for the different plans.

The discrepancies between the calculated and the measured mean LOTs were calculated for both MV detector and optical sensor methods with the thresholds measured during calibration process. Finally, the mean LOTs performed by the active leaves calculated with the optical sensor and MV detector methods were compared to one another and to the “ground truth.” Statistical correlation between the mean LOTs calculated with the optical sensor method and the measured mean LOTs as well as between the mean LOTs calculated with the MV detector method and the measured mean LOTs were studied by using the Pearson's correlation coefficient (r).

To highlight the applicability of the methods in treatment delivery analysis, the LOTs were calculated with both LOT calculation methods for the 15 fractions of a real‐case skin cancer patient. The calculated LOTs were compared to the planned ones to show the divergence between planned and calculated LOTs.

## 3 RESULTS

### 3.1 Calibration and validation of the methods

**FIGURE 3:**
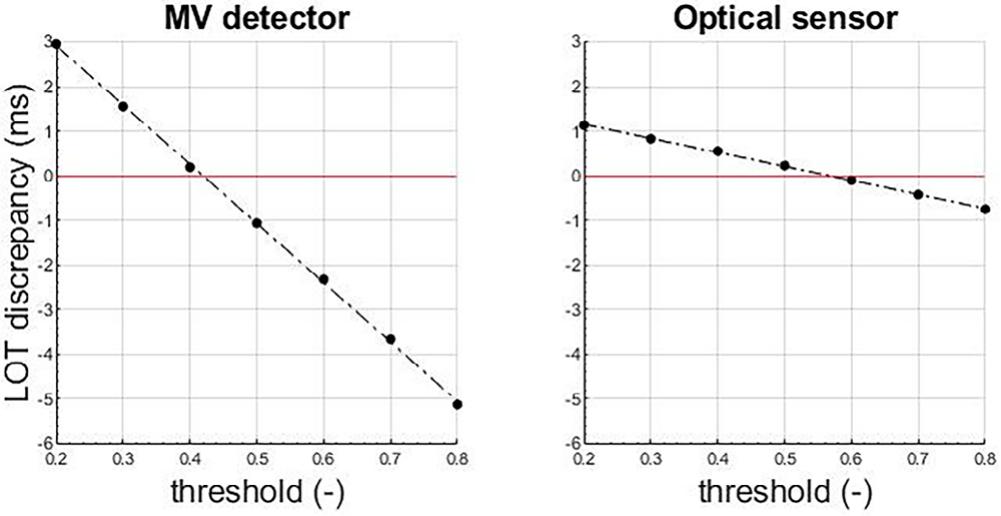
Discrepancy between calculated and measured mean LOTs when different thresholds are used for the optical sensor and the MV detector methods.

Figure 3 illustrates the mean discrepancy between the calculated mean LOTs and the measured mean LOTs when different thresholds are used for both MV detector and optical sensor methods. It shows that a mean LOT discrepancy of 0 ms is obtained for $\tau_{MV}=0.42$ (MV detector method) and $\tau_{opt}=0.57$ (optical sensor method). The LOT discrepancy is −1.1 ms for the MV detector method with $\tau_{MV}=0.50$ and 0.2 ms for the optical sensor method with $\tau_{opt}=0.50$.

**FIGURE 4:**
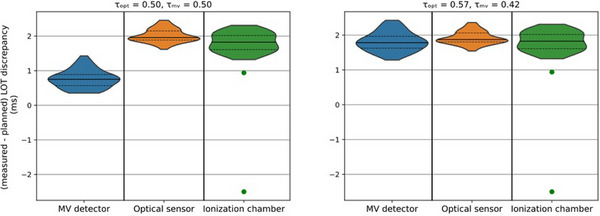
Discrepancy between the measured and the planned mean LOTs for the MV detector, the optical sensor and the ionization chamber methods.

**FIGURE 5:**
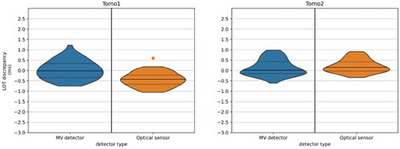
Violin plot of the LOT discrepancies between calculated (with the MV detector method in blue and the optical sensor method in orange) and measured LOTs with the ionization chamber for Tomo1 and Tomo2 devices. As the performed LOTs were calculated for the plans with planned LOTs of *x* = 25%, 50%, and 75% of the projection time, that is, for 18 plans, each violin has 18 plans * 3 active leaves = 54 data points.

Figure 4 shows the discrepancies between the mean LOTs measured with the optical sensor, MV detector and ion chamber methods, and the planned mean LOTs. The left plot shows the discrepancies when $\tau_{MV}=0.50$ and $\tau_{opt}=0.50$ are used and the right plot shows the discrepancies when $\tau_{MV}=0.42$ and $\tau_{opt}=0.57$ are used for calculations. In the left plot, the mean LOT discrepancy is 0.8 ms for the MV detector method, 2.0 ms for the optical sensor method, and 1.8 ms for the ionization chamber method. In the right plot, the mean LOT discrepancy is 1.8 ms for the MV detector and the ionization chamber methods and 1.9 ms for the optical sensor method. The uncertainties of the measured mean LOTs ranged between 0.3 and 0.5 ms. With significance level of 0.05, the results of the statistical *t*‐test show no significant differences between the mean LOT discrepancies calculated with the LOT calculation methods and the measured mean LOT discrepancies with the thresholds $\tau_{MV}=0.42\;\left(p=0.95\right)$ and $\tau_{opt}=0.57\;\left(p=0.05\right)$, while a statistically significant difference has been observed with the thresholds $\tau_{MV}=0.5\;\left(p=4\times 10^{-15}\;\right)$ and $\tau_{opt}=0.50\;\left(p=4\;\times \;10^{-3}\;\right)$. For this analysis, the two artefact outliers of −2.5 and 0.9 ms measured with the ionization chambers were removed. Figure 5 shows the discrepancies between the calculated and the measured mean LOTs for both the MV detector and the optical sensor methods using the optimal thresholds of $\tau_{MV}=0.42$ and $\tau_{opt}=0.57$. In case of the MV detector method, the discrepancies are between −0.8 and 1.2 ms. One outlier of 5.9 ms was found on Tomo1. The largest interquartile range was 0.7 ms, calculated on Tomo1. The mean discrepancy variates between 0.0 and 0.1 ms and the median is 0.0 ms for both units. The standard deviation is 0.4 ms for both units. In case of the optical sensor method, the discrepancies are between −1.1 and 0.9 ms. Two outliers of 5.9 and 0.6 ms were found on Tomo1. The largest interquartile range was 0.50 ms, calculated on Tomo1. The mean and the median discrepancies variate between −0.4 and 0.2 ms. The standard deviation was 0.4 ms for both units.

**FIGURE 6:**
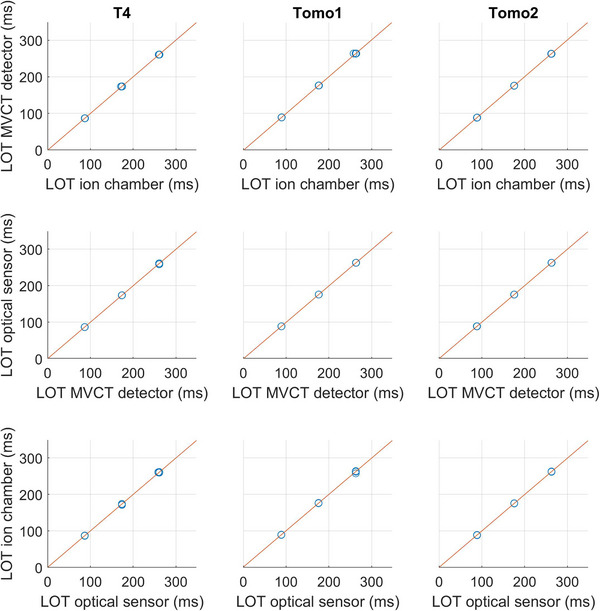
Comparison between measured and calculated LOTs for T4 (first column), Tomo1 (second column), and Tomo2 (third column). The lines are least square linear fits.

Figure 6 compares the mean LOTs calculated with the MV detector method to the ground truth (first line), the mean LOTs calculated with the optical sensor method to the mean LOTs calculated with the MV detector method (second line) and the ground truth to the mean LOTs calculated with the optical sensor method (third line). The linear fits have a slope of 1.0 and the intercepts are between −1.0 and 1.2 ms. The statistical Pearson's correlation coefficients (*r*) between the calculated mean LOTs and the measured mean LOTs for both methods were calculated along with the respective *p*‐values. The correlation coefficient being 1 across the different machines and methods indicates a perfect positive linear relationship between the calculated and the measured mean LOTs for both methods. The corresponding *p*‐values being between $3\;\times \;10^{-133}$ and $7\;\times \;10^{-104}$ indicate statistically significant correlations.

### 3.2 Example of clinical application

**FIGURE 7:**
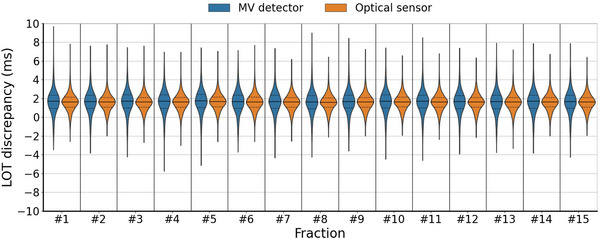
MLC performance for every fraction of a skin cancer patient.

The LOT calculation methods allow the determination of LOTs performed by the MLC in real time, during treatment deliveries, for each treatment fraction. Thus, they enable a report of the quality of the dose delivered to the patients throughout the entire course of the treatment. Figure 7 shows an example of the LOTs performed by the MLC during the delivery for a skin cancer patient. The prescription for this patient was 34.5 Gy in 15 fractions for the PTV D50%. The plan has 1251 projections with a time per projection of 0.345 s, a modulation factor of 1.899, a gantry period of 17.6 s, a pitch factor of 0.287 and a planned field width of 2.5 cm. No LOTs are below 50 ms and 48.5% are short LOTs (below 100 ms). Both methods yield consistent results for the average LOTs, median LOTs, and LOT standard deviations, with these values remaining stable across the fractions. Discrepancies in minimum and maximum values can be observed between the two methods. Systematic shifts of 1.6 and 1.7 ms in the LOT discrepancy distributions can be observed.

## 4 DISCUSSION

In this work, two methods of LOT calculation were implemented. The first method uses data collected by the MV detector and the other one uses data collected by optical sensors mounted on the MLC to calculate the LOTs. In‐phantom ionization chamber measurements were performed to calibrate the methods to the ground truth by determining the optimal thresholds $\tau_{MV}$ and $\tau_{opt}$. Additionally, in‐phantom ionization chamber measurements were performed to validate the two methods.

The goal of the measurements described in Section 2.4 was to validate and to compare the two LOT calculation methods. Even if the A1SL ionization chamber dimension is large compared to the radiation field size (ionization chamber volume is 53 mm^3^ and the smallest leaf dose profile is 6.2 × 10 mm FWHM at measurement positions), the consideration of the volume averaging correction factor is unnecessary in this study because the focus lies solely on the relative measured charge rather than the absolute charge at the measurement point.

There is no consensus on the thresholds which should be used to calculate the LOTs. Some authors defined the LOTs as the full width at half maximum ($\tau_{MV}=0.50)$ of the normalized signal^27^, ^30^ or as the sum of the products of final fluence and pulse length at each projection.^31^ Concerning the optical sensor method, Accuray Inc. uses the threshold $\tau_{opt}=0.50$. In this work, it is shown that optical sensor and MV detector methods with $\tau_{MV}=0.42$ and $\tau_{opt}=0.57$ agree with the ionization chamber measurements (Figures 4 and 5). As the uncertainties of the measured mean LOTs ranged between 0.3 and 0.5 ms, the LOTs calculated with $\tau_{\mathrm{MV}}=0.50$ are not within the uncertainties. Furthermore, there are significant differences between the calculated and the measured mean LOTs for $\tau_{MV}=0.50$ and $\tau_{opt}=0.50$, while no significant differences are observed for $\tau_{MV}=0.42$ and $\tau_{opt}=0.57$, indicating that the thresholds defined in this work are most representative of the “ground truth”. The results show a mean LOT discrepancy of 1.8 ms between the ionization chamber measurements and the planned LOTs on T4 (Figure 4). Similar results were observed on the other Radixact units, with 2.2 ms on Tomo1 and 1.5 ms on Tomo2. The discrepancy was also observed for the skin cancer patient case (Figure 7) with mean LOT discrepancies of 1.6 ms and 1.7 ms. In tomotherapy, a deviation between the planned and the actual LOTs may lead to clinically relevant dosimetric inaccuracies.^24^, ^25^ However, the dosimetric impact of the systematic shift on the treatments depends on the plan specifications, such as the jaw opening, the number of active leaves, and the LOTs, and should be evaluated case by case.

Accuray Inc. uses a LOT calculation method based on the optical sensor data with a threshold $\tau_{opt}=0.50$ for MLC performance analysis. Thus, the agreement between the calculated mean LOTs using the optical sensor method with $\tau_{opt}=0.50$ and the ionization chamber measurements validates the methodology employed by Accuray Inc. for MLC performance evaluation. On the other hand, the threshold $\tau_{MV}=0.42$ gives a better agreement between the LOTs calculated using the MV detector method and the ionization chamber measurements compared to the threshold $\tau_{MV}=0.50$ used in the work of Schopfer et al.,^28^ which enhances the method by reflecting the ground truth. The results show no considerable differences between the LOTs calculated with the MV detector method and the LOTs calculated with the optical sensor method (Figure 5). Furthermore, the correlation analysis between the calculated and the measured mean LOTs shows a perfect linear relationship between the methods and the “ground truth”. The uncertainty observed between the calculated LOTs and the ground truth is 0.4 ms for both methods. The LOTs calculation with the optical sensor data is a method validated and used by Accuray Inc. for MLC performance analysis. On the other hand, the MV detector method is an independent method. This work investigates the “independent method” developed by Schopfer et al.^28^ by demonstrating an equivalence between the method and a “company‐driven method” each relying on entirely distinct sets of information. Furthermore, the two methods allow graphical visualization of the LOT differences in a sinogram. Schopfer et al.^29^ demonstrated that the MV detector LOT calculation method enables the assessment of the LOTs performed by the MLC in‐air or in real time. Similarly, optical sensor data can be gathered for any treatment delivery, enabling the determination of the executed LOTs in‐air and in real time using the optical sensor LOT calculation approach. The optical sensor method offers an advantage over the MV detector method because it allows to make measurements for dynamic jaw deliveries when the beam is off the detector.

There is a controversy regarding the usefulness of pretreatment dose verifications in conventional radiotherapy. Furthermore, online adaptive radiotherapy workflow excludes the possibility of conducting pretreatment dose measurements. To overcome these complexities, some studies investigated the possibility of using secondary dose calculations^18^ or periodic dose measurements.^19^ The methods implemented in this study enable a continuous monitoring of the MLC performance for each treatment fraction delivery, as previously shown in the clinical example (Figure 7), without incurring additional dose to the patient. However, they do not verify other errors that may occur during delivery, such as patient misalignment, jaw positioning errors, couch, and gantry motion inaccuracies. Nevertheless, these methods have the potential to be integrated in a PSQA tool of dose delivery verification based on LOT, such as the one developed by Schopfer et al.^27^ This tool has an advantage over both secondary dose calculations, which solely assess dose calculation without verifying delivery fidelity, and periodic dose measurements, which are not conduced for every treatment fraction. While fraction‐to‐fraction monitoring has the potential to enhance PSQA across various treatment modalities by enabling verification of individual as well as overall deliveries, its significance becomes particularly pronounced in online ART because the plans may vary from one fraction to another. In this sense, the verification of the two methods implemented in this work in traditional non‐adaptive cases points to its suitability as a check of delivery fidelity in online ART cases. As both method allow in‐air and in real time MLC performance evaluation, they can be introduces in a LOT‐based PSQA tool for pretreatment dose verification, akin the conventional clinical PSQA approach, with the added benefit of being phantomless as well as in a LOT‐based approach of in real time PSQA, which allow to report the quality of the daily adapted delivered dose and to adapt for the next fractions if necessary.

One limitation of this work lies in the fact that the dose delivery used for the measurements are simple plans with static couch, gantry, and jaws and with only three active leaves. The delivery of clinical treatment plans adds more complexity to the MLC, couch, gantry, and jaws movements. Conducting ionization chamber measurements with clinical treatment plans and juxtaposing them with the calculations using MV detector and optical sensor methods would allow a global comparison of these techniques in real clinical scenarios. However, the measurements performed in this work allowed to assess the validity of both methods against the ground truth with well‐known measured LOT, which would not be possible for clinical cases where the comparison would only be related to an average of multiple LOTs. Another limitation arises from the uncertainty surrounding the suitability of a LOT‐based delivery verification tool as substitutes for PSQA measurements. Consequently, additional investigations are required to compare conventional with LOT‐based PSQA tools.

## 5 CONCLUSION

Two methods of LOT measurements for the Radixact treatment units were implemented. Their validity was assessed by comparing them with ionization chamber measurements. These techniques can find application in Adaptive Radiation Therapy (ART) by providing a means to assess the quality of the daily adapted delivered dose and facilitating adjustments for the next fraction if necessary.

## AUTHOR CONTRIBUTIONS

**Marie Nasrallah**: Conceptualization; data curation; formal analysis; investigation; methodology; resources; software; visualization; writing—original draft; writing—review & editing. **François Bochud**: Conceptualization; funding acquisition; project administration; supervision; validation; writing—review & editing. **Neelima Tellapragada**: Software; validation; writing—review & editing. **Jean Bourhis**: Resources; validation; writing—review & editing. **Edward Chao**: methodology; resources; software; visualization; writing—review & editing. **Dylan Casey**: Methodology; resources; software; visualization; writing—review & editing. **Raphaël Moeckli**: Conceptualization; funding acquisition; methodology; project administration; supervision; validation; writing—original draft; writing—review & editing.

## CONFLICT OF INTEREST STATEMENT

This research was funded by Accuray Inc. Neelima Tellapragada, Edward Chao and Dylan Casey are employees of Accuray Inc.
